# Standardizing selection criteria in nasal medication studies

**DOI:** 10.1016/S1808-8694(15)30552-8

**Published:** 2015-10-19

**Authors:** Andrei Borin, Eduardo Abib, Cleomines Izidio Araujo, Luis Lopez Martinez, Heloisio Rodrigues

**Affiliations:** 1 M.D., Ph.D, Department of Clinical Research, Libbs Pharmaceutical Ltda. Sao Paulo, SP, Brazil; 2 M.D., Ph.D, Scentryphar Clinical Research Ltda., Campinas, SP, Brazil; Medical Science School, Campinas University - UNICAMP, SP, Brazil; 3 Pharm., Department of Clinical Research, Libbs Pharmaceutical Ltda. Sao Paulo, SP, Brazil; 4 Ph.D, Department of Clinical Research, Libbs Pharmaceutical Ltda. Sao Paulo, SP, Brazil. Center of Biosciences Applied to Patients with Special Needs, CEBAPE - FOSJC, Paulista State University - UNESP, SP, Brazil; 5 M.D., Department of Clinical Research, Libbs Pharmaceutical Ltda. Sao Paulo, SP, Brazil. Scentryphar Clinical Research Ltda. Campinas, SP, Brazil. Department of Clinical Research, Libbs Pharmaceutical Ltda., Sao Paulo, SP, Brazil

**Keywords:** cytology, mucociliary clearance, control groups, nose, skin tests

## Abstract

Clinical studies on nasal topical medications require the standardization of “nasosinusal normality” in order to establish control groups through a specific evaluation of the upper airways.

**Aim:**

to standardize the evaluation of candidates for control groups in clinical studies on nasal topical medications.

**Materials and Methods:**

healthy male volunteers of 18 to 50 years of age, asymptomatic from the nasosinusal standpoint were subjected to a sequential and excluding assessment made up of clinical evaluation, immediate hypersensitivity skin test, saccharin test, flexible nasofibroscopy and nasal cytology.

**Study design:**

Cross-sectional contemporary cohort.

**Results:**

Of the 33 people originally enrolled, 14 (42.4%) were excluded for clinical reasons. Of the 19 remaining, 2 (10.5%) had atopy diagnosed in the skin test and were excluded. 17 were tested with saccharin and presented normal mucociliary clearance. Evaluation by nasal endoscopy showed abnormality in 2 cases (11.8%) and these were excluded. The remaining 15 were submitted to nasal cytology, which proved normal, representing 45.5% of those initially included.

**Conclusion:**

The proposed protocol for sequential and excluding evaluation was effective in defining candidates for the establishment of control groups in clinical studies on nasal topical medications.

## INTRODUCTION

Nasosinusal physiology is important for promoting quality of life and preventing respiratory disease, especially in large cities, where there are many aggressors to the respiratory mucosa, such as low atmospheric humidity, a high concentration of pollutants, and indiscriminate use of air conditioning systems.^1^ Diseases such as rhinitis, sinusitis, and airway viral infections reach high prevalence and morbidity rates among these populations, leading to other even more severe conditions, such as asthma, bronchitis, pneumonias, and emphysema.^2^ Topical nasal medication aims to clean and hydrate the mucosa (saline solutions,^3^, ^4^ nasal gel,^5^, ^6^, ^7^ and ringer lactate solution^8^) and to administer corticosteroids topically.^6^ It is also possible to use this route for systemic drugs, given its extensive capillary network.^9^ Controlled clinical studies are thus needed to demonstrate the efficacy and safety of drugs given by this route.

Characterizing “normal” or healthy subjects nasosinusally is important in studies of topical nasal drugs, both for PHASE 1 trials, which classically include healthy subjects, and in PHASE 2, 3 and 4 trials, in which it is necessary to confirm the nosological entity being studied and control groups.^10^ In defining a control group, it is necessary to make sure that subjects have no systemic diseases that might affect their general health; such diseases included arterial hypertension, diabetes mellitus, and chronic renal failure. Additionally, factors that may interfere pharmacologically with the drugs being studied - such as use of other medications - should be excluded.

The gender and age of subjects should also be considered, since female hormones, for instance, may affect the nasal mucosa, altering nasosinusal physiology,11 and elderly populations may present typical changes of ageing in the nasal musoca.^9^ For this reason, most of the clinical trials for evaluating topical drugs chooses adult males as their study populations, except when the study drug is specifically indicated for the female, pediatric or elderly populations. Environmental conditions may also affect such choices;^1^, ^9^, ^12^ geographically distant populations may be exposed to very different conditions of air humidity, temperature, presence of pollutants, and irritative or allergenic agents, which compounds the difficulties of standardizing control groups and characterizing a healthy nasal mucosa.^1^,^9^,^8^

Anatomical features of the nose, such as septal deviation, turbinate hypertrophy, or polyps, and a history of surgery or recent airway infections, may also interfere with nasosinusal physiology. Other factors should also be taken into account, such as smoking, medication or use of illegal drugs by a nasal route.

We conducted a survey of nasosinusal conditions of a population declared as healthy and asymptomatic, recruited at a research center in the city of Campinas, Sao Paulo state, to define a test protocol aiming at standardizing and demonstrating a status of nasosinusal health and “normalcy”, and to define selection and exclusion criteria for research subjects in clinical trials using topical nasal medication. We wrote a protocol that comprises a careful clinical evaluation, a sequential and excluding immediate hypersensitivity skin test, the saccharine test, flexible nasofibroscopy, and a nasal cytogram.

## METHOD

This study was undertaken at the Scentryphar Pesquisa Clínica Ltda research center located in the city of Campinas, Sao Paulo state, with funding from Libbs Pharmaceutical Ltd, and conforming to Brazilian and international guidelines for good clinical practices, established by the International Harmonization Conference (GCP - ICH), and also in conformity with the Helsinki Declaration principles defined by the World Medical Association (WMA). The Institutional Review Board of the Medical Science School, Campinas University - UNICAMP approved this study (number 1.046/2007). A free informed consent form was made in line with institutional requirements and applied to subjects that volunteered for this study.

Self-declared healthy, disease-free and nasosinusally asymptomatic male adults aged from 18 to 50 years were invited to participate in this study. A 5-step sequential evaluation was done, as follows:

Step 1: Clinical assessment (CA).

The following inclusion and exclusion criteria were defined in this step: inclusion criteria - healthy, male subjects aged from 18 to 50 years, with no medical history of rhinitis, sinusitis, asthma or chronic bronchitis, able to sign the free informed consent form; exclusion criteria - having participated in any other clinical trial within the past one year, a medical history of upper airway viral infection or sinusitis within the last three weeks, having used any topical nasal medication within the last four week, having taken systemic corticosteroids, antihistaminic drugs or decongestion drugs within the last four weeks, a history of nasal or sinus surgery within the last five years, smoking or a history of smoking within the last five years, daily alcohol consumption and/or having used illicit drugs within the last two years. A general physical examination was also done in this phase, including body temperature, pulse and arterial pressure measurements, and anterior rhinoscopy. Cases not encompassed by the inclusion/exclusion criteria, or with findings in the physical examination, were excluded.

Step 2: Immediate hypersensitivity skin test (HT)

Subjects selected in step 1 (CA) underwent step 2 (HT). Those positive for histamine (positive control) and negative for a 0.9% saline solution (negative control), standard acarid antigens (D. pteronyssinus, D. farinae, B tropicalis), fungi (A. alternata, C. herbarum, A. fumigatus), cockroaches (B. germanica, P. americana), animals (Cannis familiaris, Fellis domesticus) and pollen (P. pratense, L. perenne, D. glomerata, F. pratensis) were considered fit for the next step. Other findings were taken as indicating abnormalities, and such subjects were excluded.

Step 3: Saccharine test (ST).

Subjects selected in step 2 (HT) underwent the ST in a controlled environment to evaluate mucociliary clearance. Perceiving a sweet taste within 45 minutes was considered a normal result. Longer periods were considered as altered mucociliary clearance, and such subjects were excluded.

Step 4: Flexible nasofibroscopy (NF).

Subjects selected in step 3 (ST) underwent NF for an endoscopic anatomical and functional evaluation of the nasal cavities. The nasosinusal status was considered as normal when the following were absent: increased nasal discharge, altered aspect of the lower nasal turbinate, the nasal mucosa or the middle meatus, obstructive septal deviation, and nasal polyps. Other findings were considered as indicating abnormalities, and such subjects were excluded.

Step 5: Nasal cytogram (NC).

Subjects selected in step 4 (NF) underwent NC to evaluate the cells in a nasal smear. Full and percentage counts were made of epithelial cells (columnar, flat and goblet) and leukocytes (polymorphonuclear, lymphocytes and eosinophils). Analysis of the nasal cytogram was conducted at a reference laboratory (Fleury S.A).

Frame 1 shows the technical guidelines for carrying out the HT, the ST, NF, and NC.

**Frame 1:**
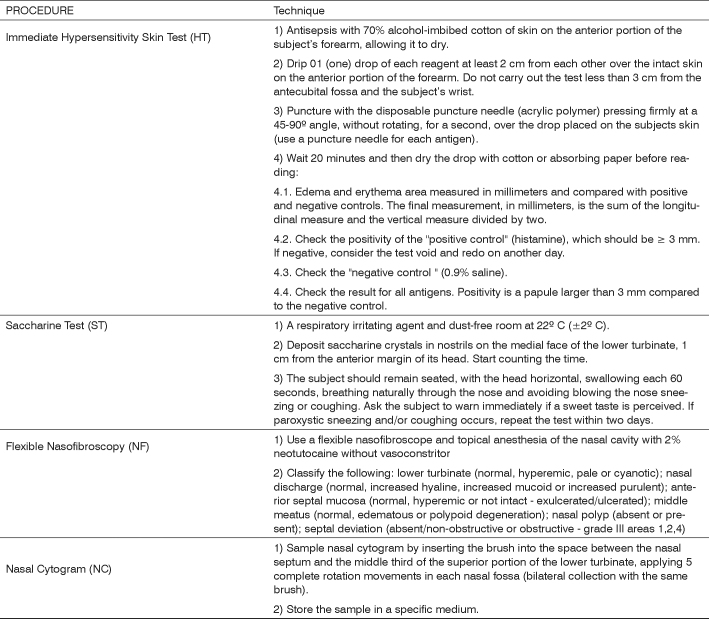
Technical guidelines for standardizing the procedures.

## RESULTS

There were 33 subjects included initially. Their mean age was 33.2 years. The CA revealed factors that could affect nasosinusal physiology in 14 of these subjects (42.4%), which were excluded (Table 1, Table 6). Of the remaining 19 subjects (57.6% of the initial number) admitted to step 2 (HT), two cases had immediate hypersensitivity to acarids (10.5%) and were excluded. No other hypersensitivity to antigens was seen in the remaining patients (Table 2).

**Table 1:** Results of the Clinical Assessment (CA).

	N	Mean	Standard deviation	Median	Minimum - Maximum
Age (years)	33	33.2	6.8	34	22.0 – 47.0
Body temperature (°C)	33	36.0	0.4	36.1	35.1 – 36.8
Pulse (bpm)	33	64.7	6.9	66	50 – 80
Weight (Kg)	33	73.3	11.1	69.3	59.5 – 97.6
Systolic pressure (mmHg)	33	125.9	10.4	126.0	103.0 – 140
Dyastolic pressure (mmHg)	33	74.6	9.0	74.0	56.0 – 900
Height (m)	33	1.71	0.07	1.70	1.60 – 1.90
Race					
Black: 8 (24,2%)			Caucasian: 25 (75,8%)

**Table 6:** Causes of exclusion by protocol phases.

	initial n	excluded	causes of exclusion
CLINICAL ASSESSMENT (CA)	33	14 * (42,4%)	turbinate hypertrophy (n=5), history of smoking within last 5 years (n=2), upper airway viral infection or sinusitis within last 3 weeks (n=2), septal deviation (n=1), use of topical nasal medication, corticosteroids or antihistamines within last 4 weeks (n=1), history of wheezing in childhood (n=1), daily consumption of alcohol (n=1), not meeting all inclusion criteria (n=1).
SKIN TEST (HT)	19	2 (10,5%)	Positive for acarids (n=2)
SACCHARINE TEST (ST)	17	0	Not excluded.
FLEXIBLE NASOFIBROSCOPY (NF)	17	2 (11,7%)	Obstructive septal deviation (n=2)
NASAL CYTOGRAM (NC)	15	0	Not excluded

**Table 2:** Results of the Immediate Hypersensitivity Skin Test (HT).

	N	positive	negative
Positive control	19	19	0
Negative control	19	0	19
D. pteronyssinus/D. farinae (ácaro 1)	19	2*	17
B. tropicalis (acarid 2)	19	1*	18
A. alternata, C. herbarum, A. fumigatus	19	0	19
B. germânica, P. americana	19	0	19
Canis familiaris	19	0	19
Fellis domesticus	19	0	19
P. pratense, L. perenne, D. glomerata, F. pratensis	19	0	19

* positive for acarids (n=2): one patient was positive for 2 antigens; another to only one

ST and NF were done in 17 subjects. The mean absolute time in the ST was 2 minutes and 54 seconds, ranging from 1 min and 8 sec to 5 min and 23 sec (Table 3). Obstructive septal deviation was found in 11.8% of subjects, which were excluded from the study (Table 4). Thus, 15 subjects underwent CN, which revealed predominantly epithelial cells and rare leukocytes (Table 5).

**Table 3:** Results of the Saccharine Test (ST).

N=17	Minutes / Seconds
Mean	2 / 54
Standard deviation	1 / 34
Median	2 / 32
Minimum - Maximum	1 / 08 – 5 / 23

**Table 4:** Results of Nasofibroscopy (NF).

	N	Absent	Present
Septal deviation	17	15	2
Nasal polyps	17	17	0
Nasal discharge	17	17	0
Altered lower turbinate	17	17	0
Altered mucosa of the anterior nasal septum	17	17	0
Altered middle meatus	17	17	0

**Table 5:** Results of the Nasal Cytogram (NC).

Epithelial cells	Numerous 11 (73.3%) Frequent 4 (26.7%)
Columnar	88.9% (± 12.7)
Flat	0.1% (± 0.3)
Goblet	11.1% (±12.7)
	Absent: 1 (6.7%)
White blood cells	Rare: 9 (60.0%)
	Few: 3 (20.0%)
	Moderate: 2 (13.3%)
Polymorphonuclear	35,0% (± 28.9)
Lymphocytes	57,3% (±32.0)
Eosinophil	0,7% (±2.0)
Mast cells	Absent: 93.3%
	Rare: 6.7%

Therefore, 15 subjects (45.5%) of the 33 subjects originally included were classified as candidates for control groups in studies of topical nasal drugs. Table 6 shows the excluding causes in various phases of the study.

## DISCUSSION

The tests and exams applied in this study to standardize the selection and exclusion criteria of research subjects for control groups in clinical trials of topical nasal drugs are well-known diagnostic procedures in the scientific literature, and have been used for safely assessing the nasal anatomy and physiology of subjects.^3^, 12, 13, 14, 15, 16, 17, 18, 19 Carrying out CA as the first selection step aimed at promptly evaluating the general health status and factors that might affect nasosinusal physiology.

HT was chosen as the second step due to the possibility that atopy might affect the compliance to topical nasal therapy, as well as more serious nasal irritation. HT was applied to check for allergen hypersensitivity; the allergens were chosen based on the literature.^12^ Our results showed that even without specific complaints or a personal history of allergy, 10.5% of our study population were positive for one or more of the antigens we tested, which justified this test.

ST has been described in the literature as a representative functional test of nasal mucociliary clearance; it correlates very well with radioisotope testing.^13^, ^14^, ^20^ Radioisotope testing is the gold standard, but is restricted to small groups because of exposure to ionizing radiation and cost.^13^, ^14^, ^20^ Thus, ST is a faster, practical and more economical option for evaluating nasal mucociliary clearance.^13^, ^14^, ^20^

Literature data on ST define a normal result when subjects report a sweet taste up to 30 minutes after the stimulus. This test is often used for screening purposes to find patients requiring more detailed assessments of mucociliary clearance.^13^ We found no case in which this parameter was altered; the mean result was below 3 minutes. Subjects undergoing this test had already been selected in previous steps (CA and HT) to investigate nasosinusal symptoms.

NF is routinely done in otorhinolaryngology; it is very sensitive and specific for evaluating the anatomy and function of the nose.^15^, ^21^ Topical anesthesia is needed for NF, which could have affected mucociliary beats and taste, which explained the sequence of procedures in this test (NF was done after ST). Our results revealed that in an asymptomatic population, about 11% of subjects had anatomical features that could have altered the application and dispersion of topical nasal drugs; underlying the importance of this test.

NC identifies the cell types in the respiratory mucosa, and is used in various clinical trials.16-18 The results revealed that our sample was within normal limits, according to the literature;^22^, ^23^ columnar cells predominated, there were few leukocytes and rare eosinophils. This results reflected the effectiveness of previous steps (CA, HT, ST and NF) for selecting “normal” subjects, since the prevalence of allergic rhinitis (10-30%) and acute rhinosinusitis (viral and bacterial) is high in the general population.^3^, ^19^ We would thus expect more inflammatory and/or allergic manifestations. NC was chosen for this step in characterizing a control group because of its importance to investigate possible local effects of study drugs. NC was the final procedure in our selection sequence, because bleeding might occur when collecting the sample, which would interfere with NF and ST.

Notwithstanding the sensitivity and specificity of these procedures, CA should be considered as a fundamental assessment tool, as it detected factors affecting nasosinusal physiology in over 40% of the asymptomatic study sample. Furthermore, the selection sequence above was practical and rapidly done; all procedures were carried out in a single visit, which avoids subjects having to return unnecessarily.

## CONCLUSION

The examination protocol consisting of carrying out a careful clinical history, an immediate hypersensitivity skin test, the saccharine test, flexible nasofibroscopy, and a nasal cytogram, in sequence and excluding subjects at each step if indicated was effective and safe for selecting nasosinusally healthy subjects. Our results suggest that this protocol may be applied for standardizing the inclusion and exclusion criteria of research subjects for control groups in clinical trials on topical nasal medication, and to define the status of baseline physiological parameters in studies assessing the response of the nasosinusal mucosa to repeated and prolonged stimuli.

## ACKNOWLEDGMENTS

The authors wish to thank Doctor Priscila Bogar Rapoport, Doctor Anete Sevciovic Grumach (Medical School of the ABC Foundation), and Doctor Ivo Bussoloti Filho (Medical Science School, Santa Casa of Sao Paulo) for medical suggestion during the design of the method.
